# *Streptococcus mitis*/human gingival fibroblasts co-culture: the best natural association in answer to the 2-hydroxyethyl methacrylate release

**DOI:** 10.1111/j.1600-0463.2011.02828.x

**Published:** 2012-02

**Authors:** Mara Di Giulio, Simonetta D'Ercole, Susi Zara, Amelia Cataldi, Luigina Cellini

**Affiliations:** 1 Department of Drug Sciences, University ‘G. d'Annunzio’Chieti-Pescara, Italy; 2 Department of Biomedical Sciences, University ‘G. d'Annunzio’Chieti-Pescara, Italy; 3 Department of Medicine and Ageing Sciences, University ‘G. d'Annunzio’Chieti-Pescara, Italy

**Keywords:** Co-culture, HEMA, human gingival fibroblasts, *Streptococcus mitis*

## Abstract

One of the major components of dental polymerized resin-based restorative materials is 2-hydroxyethyl methacrylate (HEMA) and its release in monomeric form interferes with the oral cavity environment. This study aimed to evaluate HEMA monomeric effects on the co-culture of *Streptococcus mitis* and human gingival fibroblasts (HGFs). *Streptococcus mitis* DS12 and *S. mitis* ATCC 6249 were co-cultivated with HGF in the presence of HEMA (3 mM), for 48 and 72 h; the amount of sessile and planktonic cells, as well as the prokaryotic and eukaryotic cell viability were analyzed in treated and untreated samples. The treatment of *S. mitis*/HGFs with HEMA did not produce significant effects on the bacterial adhesion and induced an increase in planktonic *S. mitis* ATCC 6249 population after 48 and 72 h. HEMA increased significantly the planktonic *S. mitis* ATCC 6249 viability when co-cultured with HGFs, while a cytotoxic effect on HGFs, without bacteria, was recorded. An increase of bacterial aggregation on HGFs was also detected with HEMA. Data obtained in this study suggest that HEMA exhibits a toxic effect mainly on eukaryotic cells and this effect can be modulated by co-cultivation with the *S. mitis* cells which, in the presence of the monomer, enhance their aggregation rate on HGFs.

The human oral cavity is constantly exposed to a wide variety of microorganisms that interact among themselves and with hard and soft tissue surfaces (1). Gingival epithelium is populated by distinct commensal bacterial species that are able to promote tight associations with host epithelial tissues, resulting in beneficial effects on the healthy oral status (2). Several studies have demonstrated that streptococci are the predominant colonizers of early enamel biofilms and, in particular, *Streptococcus mitis* is the species mainly identified; moreover, this bacterium is also isolated as the predominant species in saliva and in soft tissue surfaces (buccal mucosa, anterior vestibule, hard palate, maxillary and mandibular lips) (3–5).

Currently, an increasing number of resin-based materials are commonly used in restorative dentistry, prosthodontics and orthodontics due to their ease of handling and esthetic properties (6). Largely used resin composites are cured by light-induced polymerization of methacrylate monomers with a degree of conversion of 50–70% (7); in fact, a complete polymerization could unfortunately not be obtained because of an increase in rigidity and steric hindrance during the process. Residual non-polymerized monomers such as triethylene glycol methacrylate (TEGDMA) or 2-hydroxyethyl methacrylate (HEMA) leak from the material and diffuse into the oral cavity (8), where they could potentially be able to alter the composition and characteristics of the microbial communities colonizing the oral surface (9) and the cell functions (10). HEMA is the major component of most adhesives or primers used in dentin bonding techniques. It is used in amounts of 35–50% to reduce viscosity and its role is crucial during the dentin impregnation process of the adhesive system due to its high water affinity. This property allows HEMA to flow into the collagen network of the dentin organic matrix, favoring infiltration, preventing collagen collapse and increasing bond strengths (11).

Furthermore, HEMA shows medium cytotoxicity and its hydrophilicity and low molecular weight makes it a critical molecule in terms of biocompatibility (10).

Little experimental data are available concerning specific interactions between the products of composite resin degradation and the oral ecosystem (10, 12–15). In particular, no information is available on the effect of resin composite monomers on bacteria that currently live in the oral cavity when co-cultivated with human oral mucosa fibroblasts. In this regard, the aim of this study was to evaluate the HEMA contribution on the co-culture of *S. mitis* strains and human gingival fibroblasts (HGFs) to evaluate biological reactions that can occur during interaction among biomaterials, host tissues and oral microorganisms.

## Materials and Methods

### Bacterial strains and growth condition

The clinical strain *S. mitis* DS12 isolated from a saliva sample and the reference strain *S. mitis* ATCC 6249 were used in the present study. The strains were tested before for their adhesive properties on polystyrene surface (16). *Streptococcus mitis* species was chosen because of its biological characteristics of both beneficial commensal of oral environment and emerging opportunistic pathogen able to promote significant diseases in immunocompromised patients and interfere with oral tissue (17).

Each strain was cultured in trypticase soy broth (Oxoid, Milan, Italy) at 37 °C for 18–24 h under anaerobic atmosphere. The overnight cultures were diluted 1:10 (v/v) in Dulbecco's modified Eagle medium (DMEM; Euroclone, Milan, Italy), antibiotic and serum-free plus 1% (w/v) sucrose and refreshed for 2 h at 37 °C n thermostated shaking (160 rpm U/min) bath (Julabo SW-20 C, Milan, Italy) aerobic condition. As demonstrated previously, DMEM did not interfere with *S. mitis* growth (18). The broth cultures were adjusted to 0.5 McFarland, corresponding to approximately 1.5 × 10^8^ CFU/mL for *S. mitis* ATCC 6249 and 1.2 × 10^8^ CFU/mL for *S. mitis* DS12 and were used for the experiments. These inocula size were similar to those used in other co-culture assays (18, 19).

### Culture of HGFs

HGFs were obtained from fragments of healthy marginal gingival tissue of one donor, from the retromolar area taken during surgical extraction of impacted third molars. Written informed consent was obtained according to a protocol approved by the University of Bologna (Italy). HGFs were used since, in the oral cavity, they are in close proximity to restorative dental materials. The tissue fragment was cultured in DMEM/F12 (Euroclone) for at least 1 h, rinsed three times in phosphate-buffered saline solution (PBS), minced into small tissue pieces and cultured in DMEM/F12 containing 10% fetal bovine serum (FBS; PAA, Pasching, Austria) and antibiotics (1% penicillin and streptomycin, 1% fungizone; Euroclone) in 125-cm^2^ flasks (SPL Life Sciences, Milan, Italy). All cells were maintained at 37 °C in a humidified atmosphere of 5% (v/v) CO_2_. Cultured HGFs at passage numbers between 13 and 18 were used for these experiments.

### HEMA treatment

HEMA was purchased from Sigma-Aldrich (Milan, Italy), and a stock solution of 1 M in ethanol was prepared and filtered through 0.2-μm pore size filters. The stock solution was then diluted in DMEM to obtain a medium containing HEMA in a concentration of 3 mM according to Falconi et al. (15). In all the incubation media, the final ethanol concentration was lower than 0.3%.

### Co-culture preparation

The co-culture was performed in microtiter plates (tissue-culture-treated plates; Nunc, EuroClone SpA, Life Sciences Division, Milan, Italy). The HGFs were seeded in microtiter plates in DMEM containing 10% FBS and antibiotics (1% penicillin and streptomycin, 1% fungizone) in a humidified atmosphere of 5% (v/v) CO_2_ at 37 °C. When cells reached confluence, the culture medium was removed and the wells were washed with PBS. The standardized bacterial cultures in DMEM 1% sucrose were then added to the HGFs together with HEMA, at a final concentration of 3 mM. Control co-cultures received medium without HEMA. In addition, in the experimental design, *Streptococcus* strains and HGFs were also assayed alone in DMEM 1% sucrose with and without HEMA. The plates were incubated for 48 and 72 h in a humidified atmosphere of 5% (v/v) CO_2_ at 37 °C. The experimental design was carried out for three independent experiments and each experiment was performed in triplicate.

### Adhesion assay

The effect of HEMA on the streptococcal adhesion both on HGFs and on polystyrene surface was evaluated on 96-well flat-bottomed microtiter plates and was quantified by safranin staining by using a modified method of Cramton et al. (20) Briefly, the planktonic phases were removed by aspiration and each well was washed twice with PBS, dried and stained with a 0.25% safranin solution (Biolife, Milan, Italy) for 1 min and then washed twice with water. The optical density (OD) of stained biofilms was measured by spectrophotometer at 492 nm using an enzyme-linked immune-adsorbent assay (ELISA) reader (SAFAS, Munich, Germany) both for the evaluation of HEMA's effect on streptococcal adhesion on polystyrene surface and on HGFs. Moreover, the removed planktonic phase coming from all the examined samples was also quantified by measuring the OD at 600 nm (OD_600_) to also detect the effect of HEMA on the unattached bacterial population. Each assay was performed in triplicate for three independent experiments.

The effect of HEMA exposure was assessed both on planktonic and sessile *S. mitis* populations to evaluate its possible different action on bacteria in unlike physiological status.

### Viability test and microscopic observations

The streptococcal sessile phases both on HGFs and on polystyrene surface and the correspondent planktonic phases were examined for their viability. After the incubation of all cultures for 48 and 72 h in a humidified atmosphere of 5% (v/v) CO_2_ at 37 °C, the planktonic phase was removed by aspiration and each well was washed twice with PBS; then, both the unattached and attached bacteria were examined for their viability with Live/Dead Kit (Molecular Probes Inc., Invitrogen, Italy) as indicated by the manufacturer and visualized under a fluorescent Leika 4000 DM microscope (Leica microsystems Spa, Milan, Italy). Ten fields of view randomly chosen for each slide were examined. The counts were repeated independently by three microbiologists by using image analysis software (LEICA QWin, Milan, Italy). Microscopic observations were repeated for three independent experiments.

### Trypan blue dye exclusion test and light microscopic analysis

The death rate of HGF cells was assessed on six-well flat-bottomed microtiter plates using the Trypan blue exclusion assay technique that measures cell membrane integrity. After 48 and 72 h, for each experimental condition, the HGFs were retrieved with a solution of 0.25% trypsin supplemented with 1 mM EDTA (Sigma-Aldrich). Aliquots of harvested cells from each well were mixed with 0.5% Trypan blue dye (Sigma-Aldrich), in a 1:1 ratio. The number of dead and living cells was determined for each condition by means of a hemocytometer (Hausser Scientific) with an optical microscope (Leica microsystems Spa, Milan, Italy) at ×100. To corroborate the data obtained, microscopic observations were made with an inverted microscope (Leica microsystems Spa, Milan, Italy) and pictures with a digital camera were taken at ×100. The Trypan blue data were presented as the mean (±SD), of triplicate experiments.

### Statistical analysis

The significance of the differences recorded in the experiments performed with and without HEMA in each experimental condition tested was evaluated using Student's *t*-test. Probability levels <0.05 were considered statistically significant.

## Results

The effects of HEMA on sessile and planktonic growth phases of *S. mitis* DS12 and *S. mitis* ATCC 6249 alone and co-cultured on HGFs were evaluated.

**Fig. 1:**
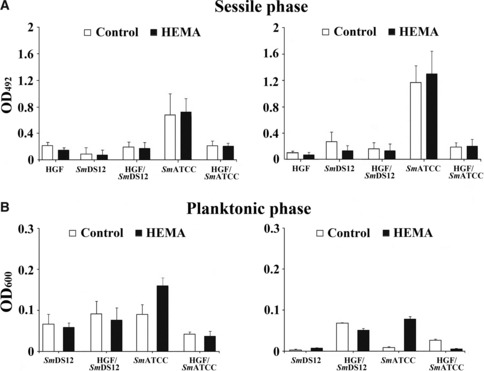
Effect of 3 mM HEMA concentration on sessile (A) and planktonic (B) growth phases of *Streptococcus mitis* DS12 (*Sm*DS12) and *S. mitis* ATCC 6249 (*Sm*ATCC) alone and co-cultured on human gingival fibroblasts after 48 and 72 h of treatment.

No significant differences in absorbance values (OD_492_) on the growth in the sessile phase of co-cultured *S. mitis* strains and HGFs at 48 and 72 h with and without HEMA were detected (Fig. 1A). When fibroblasts and bacteria were tested alone, the monomer seemed to reduce their adhesion on the polystyrene surface except for *S. mitis* ATCC 6249. In this case, the presence of HEMA on culture in DMEM 1% sucrose favored the adhesion of the reference *S. mitis* ATCC 6249 strain to the polystyrene surface both at 48 and 72 h. Furthermore, a significant increase of *S. mitis* ATCC 6249 bacterial population (p < 0.05) was also detected in the planktonic growth phase after 48 and 72 h of treatment with HEMA (Fig. 1B) with respect to the corresponding control. For the planktonic bacterial phase, the presence of HEMA displayed a reduction in OD_600_ (Fig. 1B) that was not significant (p > 0.05) with respect to the controls.

The cytotoxic effects of HEMA on the HGFs are shown in Table 1. The cell death, assessed by Trypan blue, was more pronounced when HGFs were treated with HEMA in absence of microorganisms, both at 48 and 72 h (47.3% and 46.5%, respectively); in fact, the co-cultivation of *S. mitis* strains/HGFs produced a decrease in the rate of HGFs death, both at 48 and 72 h.

**Table 1:** Percentage of cell death assessed by Trypan blue dye exclusion test, after 48 and 72 h of treatment with 3 mM HEMA. Data are the mean (±SD) of three different experiments

Culture condition	Percentage of dead cells	
	
	48 h	72 h
HGF	8.1 ± 0.4	8.4 ± 0.4
HGF/HEMA	47.3 ± 3.1	46.5 ± 2.6
HGF/*S. mitis* DS12	7.5 ± 0.5	9.2 ± 0.8
HGF/*S. mitis* DS12/HEMA	33.3 ± 2.7	30.8 ± 2.2
HGF/*S. mitis* ATCC 6249	9.2 ± 0.8	13.6 ± 1.0
HGF/*S. mitis* ATCC 6249/HEMA	28.2 ± 2.4	30.0 ± 3.0

HGF, human gingival fibroblast; HEMA, 2-hydroxyethyl methacrylate; *S. mitis*, *Streptococcus mitis.*

**Fig. 2:**
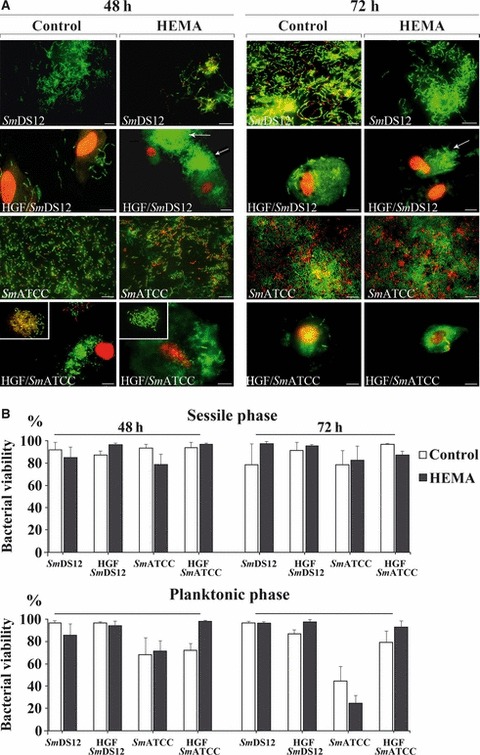
Effect of 3 mM 2-hydroxyethyl methacrylate (HEMA) concentration on the viability of *Streptococcus mitis* DS12 (*Sm*DS12) and *S. mitis* ATCC 6249 (*Sm*ATCC) alone and co-cultured on human gingival fibroblasts (HGFs) after 48 and 72 h of treatment. (A) Representative images of sessile streptococcal strains alone (rows 1 and 3) and co-cultured with HGFs (rows 2 and 4) untreated and treated with HEMA. Arrows indicate the bacterial aggregation on HGFs. Images showed in inserts represent the corresponding planktonic phases in which the HEMA treatment produces a significant increase of bacterial viability with respect to the control (see histograms; B). Live/dead staining, scale bar = 10 μm; (B) percentage of streptococcal viability on sessile and planktonic growth phases, untreated and treated with HEMA.

The HEMA effect on the *S. mitis* viability is shown in Fig. 2. No significant differences were recorded on the viability of the *S. mitis* DS12 clinical strain alone or co-cultured with HGFs after exposed to HEMA twice both in the sessile and planktonic growth phases (Fig. 2B) with respect to the controls. Although no viability modification was detected, interestingly the HEMA exposure appeared to raise the *S. mitis* DS12 aggregation and the adhesion on fibroblasts in the sessile growth phase (Fig. 2A, row 2). The analysis of the *S. mitis* ATCC 6249 viability, without HGFs co-culture, displayed a significant reduction of live bacteria (p < 0.05) in the sessile growth phase after 48 h of treatment and in the planktonic growth phase after 72 h of treatment, with respect to the controls (Fig. 2A, row 3; Fig. 2B). However, when co-cultivated with HGFs, *S. mitis* ATCC 6249 showed a significant (p < 0.05) increase of viability of free cells after 48 and 72 h of treatment (Fig. 2A, row 4 inserts; Fig. 2B).

## Discussion

Previous studies have suggested that HEMA can be released from restorative materials and it can diffuse into the oral cavity over time (15, 21). In addition to this, it is reported that resin composite extracts could have a potential impact on the ecology of oral microorganisms and induce a proliferation of caries-associated bacteria as well as induce adverse biological effects on oral mucosa (9, 10). There are several studies describing the effect of resin composite monomers on oral eukaryotic and bacterial cells separately (10, 12–15, 22), but no information is available concerning the action on both cells in a co-cultivation model. In this study, we used HGFs that are the major constituents of gingival connective tissue (23) and in particular, that are in close proximity to restorative dental materials. Moreover, *S. mitis* strains usually found as commensal of human oral environment can exert a strong immunomodulatory effect on human cells. (24). We used 3 mM concentration of HEMA according to Falconi et al. (15) that defined the right work concentration being the less toxic for eukaryotic cells and that was enclosed into the range values of HEMA release from polymerized dental adhesives (21). Finally, we used two treatment time periods (48 and 72 h) as the release of resin-based materials occurs for long periods and the cytotoxic effects increase with time of exposure (8, 25).

In this study, HEMA did not produce relevant effects on the *S. mitis* adhesion capability both on HGFs and polystyrene surface. In fact, at each time of exposure, the OD_492_ absorbance values among bacteria alone or co-cultured with HGFs with respect to the controls, were similar. The analysis of the low adhesive strain, *S. mitis* DS12 clearly revealed no effects of the monomer treatment in time. In the case of the reference strain *S. mitis* ATCC 6249 that expresses more adhesive capability, HEMA seemed to increase this property, when the strain was cultivated alone, but these values were not significant. Moreover, the presence of HEMA favored the growth of this microorganism as demonstrated by the significant increase of cells in the planktonic phase.

From these data, a possible HEMA effect as a growth factor could be supposed according to other studies that demonstrated the stimulation of oral bacterial growth by resin composite co-monomers (12, 13, 26). These authors found an increase of the total growth of oral bacteria in the presence of released biodegradation by-products. In this study, the effect of HEMA on the growth in the planktonic phase was modulated by the presence of fibroblasts. In fact, co-culture of *S. mitis* ATCC 6249/HGFs showed a reduction of bacterial growth probably due to a competitiveness for the culture medium factors or, as suggested by Johansson et al. (27) for the interaction between fibroblasts and bacteria that may be part of the regulatory mechanisms of the oral microflora during the colonization of the oral mucosa.

Moreover, Johansson et al. (27) reported that gingival fibroblasts release factors in the medium that significantly reduce growth of some bacterial species. When the HEMA effect was evaluated for the streptococcal viability, data from the present study showed that, generally, the presence of HGFs improved the *S. mitis* viability rate. When *S. mitis* ATCC 6249 was co-cultured with HGFs, in the presence of HEMA, bacteria were protected by fibroblasts from the killing effect of the monomer; in fact, HEMA produced a progressive loss of viability among *S. mitis* ATCC 6249 free cells when cultivated alone. Another valuable HEMA effect was an increase of aggregation among bacteria that was clearly evidenced in both examined strains of *S. mitis*. On the other hand, Ochiai et al. (28), demonstrated that *S. mitis* exhibits homotypic aggregation related to cell-to-cell interactions between the adhesin receptor complementary pairs on the two bacterial surfaces and, probably, the resin-based monomer emphasizes this co-aggregative effect. The human fibroblasts also profited from beneficial effects when co-cultured with *S. mitis* in the presence of HEMA. In fact, the cytotoxic HEMA effect against HGFs was reduced when fibroblasts were co-cultured with these bacteria. Therefore, the co-cultivation produced a sort of mutual protection of *S. mitis*/HGFs with a significant reduction of eukaryotic cell death. Probably, a clear relationship was established between HGFs and *S. mitis* ATCC 6249 that attempted to modulate the interactions with HEMA to benefit each other. The interaction of oral host cell and bacterium contributes to the maintenance of balance between bacteria and environment that HEMA, instead, modifies when it acts on single oral elements. Finally, from our data we can assert that there is a cross-protection between HGF and *S. mitis* strains. This confirms, as reported by several authors (2, 29), that the early phase of bacterial adhesion forming biofilm on the oral cavity has protective effects for oral tissues toward external factors such as resin-based materials and this colonization may influence the microbial succession that occurs in human dental plaque and may contribute to various disease states including gingivitis, root surface caries and periodontis. Moreover, Roberts and Darveau (2) demonstrated that associations between host tissue and oral bacteria may have beneficial effects on healthy oral status.

Further studies will be useful to elucidate the interactions among several oral components by using saliva in a co-culture model with a mixed culture of cariogenic microorganisms to reproduce the oral environment.
